# Intelligence

**Published:** 1833-02

**Authors:** 


### INTELLIGENCE.

For the following account of the case of the so-called "Lavinia or Eliza Edwards," which has lately excited so much attention, we are indebted to Mr. A. S. Taylor.

During the last month, a case has occurred of a somewhat novel nature in the annals of imposture, which has greatly excited the curiosity both of the public and of the profession: on this account, its details may not prove uninteresting to the readers of the London Medical and Physical Journal.

It appears that, a short time since, the body of an individual, professing to be that of a female of the name of Eliza Edwards, was sent to the workhouse of St. Margaret's, Westminster, for interment. It was brought from a low brothel in the neighbourhood; but no particular history was given of the deceased, and the body, being considered among the "unclaimed" of the parish, was, by order of the inspector for London, sent to Guy's Hospital for dissection. The certificate represented it to be that of a female, and it was received by the keeper of the dissecting room as such; but, a few hours after its admission, it was discovered to be that of the body of a full-grown male. Notice was given, as speedily as possible, to the authorities of the parish of St. Margaret's, in order to rectify any supposed mistake; and, in the mean time, no prohibition existing to the contrary, the dissection was continued by those gentlemen to whom the body was assigned.

An order was afterwards issued by the inspector for its removal, and an inquest was held on the body, in consequence of some suspicion being raised respecting the real cause of the death of this individual. Many extraordinary particulars regarding the life and habits of the deceased now came to light. It appeared that he had first assumed female attire about the age of fourteen; and, having chosen a theatrical life, he represented female characters on the stage, first in Paris, and subsequently in the theatres of many of the great provincial towns of England. No suspicion seems to have arisen respecting his sex, and he passed off with the public as a female. There is no doubt that many were acquainted with the real history of this impostor, but the secret seems to have been maintained most inviolably: indeed, it was only in consequence of some letters having been accidentally found in his bedroom, after death, that so much of his history has become known to the public.

A short time previously to his death, he had been attended by a physician for disease of the lungs, and so accurately was the imposition maintained that his medical attendant did not entertain a suspicion of the real sex of his patient. He had been sent as a female, when dead, by the parties with whom he lived, to the workhouse; he had been there received as such, and his body afterwards conveyed to the hospital, where the imposture was first detected. These facts have led many to believe that it was a case of sexual ambiguity; but, so far from this, a simple inspection of the exterior of the body would have sufficed to remove all doubts upon the subject. The features were somewhat of a feminine character; the hair was long, and, like that of a female, parted in the middle of the forehead. These were the only points in which any resemblance to the other sex could be traced. A great part of the beard and whiskers had evidently been plucked out, and the hair which grew under the chin had been dexterously concealed by the peculiar head-dress which he wore during life. The deceased was about twenty-four years of age at the time of death; and it appears that his voice had been remarked, by several witnesses, to be hoarse and peculiar, though not unlike that of many females at the middle period of life.

On the 24th January I was called upon officially to examine the body of the deceased, in order to ascertain the cause of death. With the exception of the appearances already described, it had every character of that of a full-grown male. There were no mammæ, the male sexual organs were perfectly developed, and the lower extremities exhibited those prominences of the muscles and bones characteristic of the male sex. The upper extremities were somewhat slender and tapering; and the hands had evidently been unused to hard manual labour. Having laid open the thorax, the lungs were found considerably diseased, and adhering to the internal parietes of that cavity; they felt hard and tuberculated, and, upon making a section through each, it presented a series of small abscesses, filled with matter. There was scarcely a portion of the organs which could be termed healthy. The pericardium contained a larger quantity of serous fluid than usual; but the heart was free from any appearance of disease, although somewhat large.

Upon opening the abdomen, the liver was found greatly enlarged, of a pale nutmeg colour, and presenting the appearance commonly observed in the bodies of individuals who have been addicted to the use of spirituous liquors. The stomach was distended with about a pint of fluid, presenting nothing remarkable in smell or colour: its coats, both internally and externally, were healthy. No trace of inflammation could be perceived in any part of the alimentary canal, nor was there any poison to be discovered in the fluid contents of the viscera. Indeed, the suspicion of poisoning was clearly negatived by the history of the symptoms immediately preceding the death of the deceased; and by the fact of his having been attended to the last by a physician who prescribed for him and watched the progress of his case. The other viscera of the abdomen were found in a healthy state; the internal organs of generation were perfect, and the contents of the pelvis presented no ambiguity either in form or disposition.

It was the opinion of those who were present at the examination, that the deceased had been living for nine years in the lowest state of infamy, and that he had probably assumed and maintained the disguise of a female, in order the more effectually to conceal his nefarious practices.

Before the coroner it was simply stated, that he had died from disease of the lungs; and, after some irrelevant evidence had been gone into, the jury found a verdict accordingly.

---

### ROYAL SOCIETY.

*Rumford Medals.* EIGHT years have now elapsed since these medals were awarded, no discovery having been made, during that period, which appeared to the trustees to deserve these high rewards: we are, therefore, happy to find that the Royal Society has now adjudged them to Professor DANIELL. It will be recollected that the Rumford medals were founded by Count RUMFORD for the most important discovery, or useful improvement, in any part of Europe, on Heat or on Light. They are directed to be adjudged every two years; and when no new discovery has taken place, during the preceding period, of importance enough to deserve the premium, the value of it is laid out in the purchase of stock, to augment the original bequest. The premium is directed to be given in two medals, struck in the same die, the one of gold, the other of silver, both of them together of the intrinsic value of £60. They have been awarded as follow: 1800, to Count Rumford; 1804, Professor Leslie; 1806, Mr. Murdoch; 1810, M. Malus; 1814, Dr. Wells; 1816, Sir H. Davy; 1818, Dr. Brewster; 1824, M. Fusnel; 1832, Professor Daniell, of King's College, London, for two papers published in the Philosophical Transactions, "on a new Register Pyrometer, for measuring the Expansion of Solids, and determining the higher Degrees of Temperature upon the common Thermometer Scale."

---

### ROYAL INSTITUTION.

THE first general meeting of the members of this Institution took place on Friday evening, the 25th January, when a very numerous auditory assembled to hear Professor BRANDE's observations on the newly-proposed system of Chemical Notation, introduced by BERZELIUS, and modified by himself. If such peculiar notations are to be adopted, Professor Brande's is much the most simple and perspicuous scheme; but, as we are persuaded that such cryptographies tend more to retard than to advance the science, we trust they will remain, at least in our schools, merely as philosophic curiosities. The most interesting communication, however, was one that, although not publicly made, is by this time too publicly known to require silence on our part, viz. the munificent patronage bestowed on science in general, and this Institution in particular, by Mr. FULLER: for, not contented with founding the Fullerian Medals for the encouragement of chemical science, this gentleman is now on the eve of endowing a Professorship of Chemistry in the Royal Institution, and, with sound discretion, has selected Dr. FARADAY to be the first Fullerian professor. This, however, is not the whole of this gentleman's liberality, although it is all we are at liberty now to communicate. We shall shortly have to record another nearly similar act.

The following are the arrangements for the season:

Evening meetings, every Friday at half-past eight P.M.

Lectures, Tuesdays and Saturdays, at three P.M.

On the Chemistry of Air and Water, by W. T. BRANDE, F.R.S.

On Heat, and the Mechanical Properties of Elastic Fluids, Steam, and the Steam-engine, by the Rev. W. RITCHIE, LL.D. F.R.S. &c.

On Geology, by CHARLES LYELL, Esq. F.R.S., Professor of Geology K.C.L.

On Electricity and Magnetism, by M. FARADAY, Esq. D.C.L. F.R.S. &c.

On Botany, by GILBERT T. BURNETT, F.L.S., Professor of Botany, K.C.L.

---

### COLLEGE OF PHYSICIANS.

The evening meetings for the season commenced on Monday, the 28th January. The Lord Chancellor, the Bishop of London, and many other distinguished persons, were present; M. CLOT BEY also attended. Sir HENRY HALFORD read a brief paper on Insanity.

### KING'S COLLEGE, LONDON.

THE students of Law and Medicine have established Societies for mutual improvement in this College, which are carried on with great spirit. Three very interesting papers have been read at the latter (which the most concerns us): one, "on Wounds in Dissection," by Mr. PARKE; another, "on Medical Electricity," by Mr. COX; and the third, "on Variola," by Mr. GOOLDEN.

---

### ACADEMIE DES SCIENCES.

DR. ROUSSEAU, whose essay on the Preparation of Ilicine and the use of that proximate principle, and of Holly, in the cure of Intermittent and Remittent Fevers, we published in our Journal for August 1832, among the Transactions of the Medico-Botanical Society, has lately had awarded to him, by the Academy of Sciences, the gold medal, worth 1500 francs. It will be remembered that, for the same discovery, he had previously received the silver medal from the Medico-Botanical Society, the members of which were only prevented awarding him their gold medal by having offered it for a different inquiry. Our opinion of the value of this discovery is fully borne out by the report made by MAGENDIE, who tried it largely in a great number of cases of ague under his care in the Hôtel Dieu; and, as he observes, Dr. Rousseau well merits the prize, "for having added to the materia medica an indigenous remedy, which will be found to be of the greatest value wherever agues are endemic, and the natives poor."

We may take this opportunity of drawing the attention of our readers to the importance of the papers which are read before this Society, for the publication of which we have received the permission of the President and Council.

---

### ZOOLOGICAL SOCIETY.

*Lectures on Comparative Anatomy.* By Dr. GRANT, Professor of Comparative Anatomy and Zoology to the University of London.

THE study of comparative anatomy and zoology, although it has conferred the highest reputation upon the names of Cuvier, Geoffroy St. Hilaire, Blainville, and other foreign anatomists, has unfortunately been very little pursued in this country; and, if some of our British physiologists have been led, for a time, to make investigations in this branch of science, they have, in most instances, hastily given up the pursuit, lest it should injure their success in the more lucrative practice of their profession. It might have been suggested to them, however, that the fame of Hunter has, perhaps, derived its greatest lustre from his investigations in comparative anatomy and animal physiology; and the museum at the College of Surgeons affords the most splendid monument of his industry and research.

The Zoological Society have begun to emerge from the state of mere showmen, and to evince some disposition to assert their rank as a scientific body, by constituting their scientific meetings on a more liberal footing, having reorganised them on the basis of other learned bodies.

Dr. GRANT, professor of comparative anatomy and zoology in the University of London, has commenced a course of lectures on the Structure and Classification of Animals, to members of the Zoological Society, at their house in Bruton street, which are to be continued every Tuesday and Thursday evenings; and a more interesting course has never been given on the subject. Profound, solid, and scientific in their general character, the learned professor is sometimes carried away by his enthusiasm for the subject, and gives occasionally bursts of eloquent description which astonish and delight his hearers: his explanation of the general characters of the class Aves, and his particular characteristics of the genus Brinana, will fully justify the warmest encomiums.

In fine, these lectures bid fair to give a new impulse to the study of natural history in this country.

*Important to Medical Pupils studying for the Army. New Army Regulations in regard to Natural History and Botany.*

We understand, by a communication just transmitted to the medical professors in the University, that candidates for the medical department of the army are required, before examination, to produce certificates of having attended a three-months' course of lectures on natural history; and another, of equal extent, on botany, at established schools of eminence.—*Edinburgh New Philos. Journal.*

### OBITUARY.

### METEOROLOGICAL REGISTER,

By Messrs. Harris and Co. Mathematical Instrument Makers, 50, High Holborn.

| Dec. | Moon. | Rain gauge. | Thermometer. | | | Barometer. | | De Luc's Hygrometer. | | Winds. | | Atmospheric Variation. | | |
|---|---|---|---|---|---|---|---|---|---|---|---|---|---|---|
| | | | 9a.m. | max. | min. | 9a.m. | 10p.m. | 9a.m. | 10p.m. | 9 a.m. | 10p.m. | 9 a.m. | 2 p.m. | 10 p.m. |
| 20 | | | 36 | 43 | 35 | 29.72 | 29.80 | 75 | 75 | NW | W | foggy | cloudy | foggy |
| 21 | | | 43 | 48 | 42 | .54 | .53 | 77 | 79 | SW | W | showery | foggy | |
| 22 | ● | .09 | 46 | 49 | 46 | .63 | .62 | 80 | 80 | WSW | SW | foggy | | |
| 23 | | | 49 | 53 | 45 | .59 | .74 | 82 | 83 | SSW | SW | rain | | |
| 24 | | | 45 | 49 | 43 | .82 | .71 | 82 | 80 | WSW | SW | foggy | fine | fine |
| 25 | | | 48 | 54 | 39 | .78 | .86 | 80 | 79 | SW | SW | rain | showery | fine |
| 26 | | | 40 | 45 | 36 | .93 | .96 | 77 | 79 | WSW | WSW | foggy | cloudy | |
| 27 | | | 38 | 44 | 33 | .96 | .96 | 79 | 80 | W | WNW | | fine | fog |
| 28 | | | 35 | 42 | 34 | .86 | .80 | 80 | 81 | N | SE | fog | fog | cloudy |
| 29 | | | 40 | 42 | 36 | .55 | .53 | 82 | 82 | SE | SE | fog | rain | sleet |
| 30 | ☽ | | 39 | 41 | 34 | .47 | .42 | 80 | 78 | SE | SE | fine | fine | cloudy |
| 31 | | | 37 | 40 | 35 | .63 | 30.02 | 78 | 80 | W | N | foggy | fog | |
| *Jan.* | | | | | | | | | | | | | | |
| 1 | | | 36 | 42 | 35 | 30.23 | .21 | 80 | 83 | WNW | WSW | | cloudy | rain |
| 2 | | .51 | 45 | 47 | 41 | 29.88 | 29.92 | 82 | 84 | SW | N | | | sleet |
| 3 | | | 42 | 44 | 36 | 30.23 | 30.33 | 84 | 80 | E | NNE | | | fine |
| 4 | | | 37 | 39 | 32 | .36 | .29 | 77 | 76 | ENE | NE | fine | fine | |
| 5 | | | 35 | 39 | 32 | .14 | .14 | 76 | 75 | NE | SE | | | |
| 6 | ○ | | 33 | 33 | 30 | .26 | .29 | 75 | 75 | SW | W | | | |
| 7 | | | 32 | 36 | 33 | .36 | .41 | 76 | 75 | W | NW | foggy | fine | |
| 8 | | | 36 | 39 | 31 | .45 | .46 | 75 | 76 | N | E | fog | cloudy | |
| 9 | | | 35 | 36 | 30 | .36 | .29 | 76 | 77 | SE | SE | | fine | |
| 10 | | | 30 | 35 | 30 | .05 | 29.89 | 77 | 79 | SSE | SSE | | | |
| 11 | | | 33 | 39 | 32 | 29.72 | .63 | 79 | 80 | SSE | SE | | | foggy |
| 12 | ☾ | | 36 | 40 | 36 | .66 | .67 | 80 | 80 | SE | SE | foggy | | cloudy |
| 13 | | | 40 | 43 | 37 | .84 | .96 | 82 | 82 | E | E | cloudy | cloudy | foggy |
| 14 | | | 41 | 43 | 37 | 30.02 | 30.02 | 85 | 83 | ESE | SE | foggy | fine | fine |
| 15 | | | 40 | 41 | 38 | 29.97 | 29.97 | 82 | 79 | ESE | SE | fine | | |
| 16 | | | 40 | 42 | 37 | 30.00 | 30.02 | 77 | 79 | ESE | E | | sleet | cloudy |
| 17 | | | 40 | 42 | 38 | 29.93 | 29.88 | 79 | 80 | E | ENE | fog | cloudy | fine |
| 18 | | .05 | 38 | 40 | 36 | .88 | .97 | 80 | 78 | NE | E | | cloudy | mist |
| 19 | | | 37 | 39 | 34 | 30.07 | 30.07 | 76 | 75 | ESE | ESE | | fine | sleet |

The quantity of Rain fallen in December, was 71.100ths of an inch.

